# Caenorhabditis elegans populations shape their microbial environment

**DOI:** 10.1038/s41522-026-00975-z

**Published:** 2026-04-04

**Authors:** Rahul Bodkhe, Kris Sankaran, Michael Shapira

**Affiliations:** 1 Department of Integrative Biology University of California, Berkeley, CA USA; 2 Department of Statistics University of Wisconsin, Madison, WI USA

**Keywords:** Ecology, Ecology, Microbiology

## Abstract

Nematodes represent one of the most abundant and ecologically significant taxonomic groups on earth, playing diverse roles in the cycling of organic matter. However, little is known about their effects on their microbial environment. To explore such effects, we took advantage of the bacteriovore free-living nematode *Caenorhabditis elegans*, which has been shown to assemble a characteristic gut microbiome from different microbial environments. Worm populations (initially germ-free) were raised in several microbially-distinct natural-like environments emulating the environment from which *C. elegans* are often isolated, allowing worms to go through four generations encompassing the typical boom-to-bust population growth cycle. Samples from worms, their environments, and from control environments without worms were analyzed using next-generation 16S rRNA gene sequencing. Data analysis showed that microbial diversity increased in the environment, either when worms were present or not, but that trajectories of change were different depending on the presence of worms. Importantly, the presence of worms led with time to convergence in the composition of their microbial environments, particularly affecting the abundance of members of bacterial families that are part of the *C. elegans* gut microbiome. Our findings reveal that *C. elegans* not only responds to environmental microbial changes but also shapes them, suggesting new roles for nematodes in modulating environmental microbial diversity and ecosystems.

## Introduction

Nematodes are among the most abundant taxonomical groups on earth, with around 28,000 described species, of which 35% are terrestrial and are thought to play crucial roles in organic matter cycling^1–3^. Their development includes four larval stages, L1–L4 in free-living nematodes and J1–J4 in plant and entomopathogenic species^4^. Many nematodes can respond to environmental conditions by entering diapause-like states, including the well-studied dauer stage in *Caenorhabditis elegans*^5,6^. A considerable attention is given to the role of nematodes in nutrient recycling in the environment, but little is known about their effects on the microbial diversity in their environment. This is of particular interest given that bacterivorous nematodes are the most abundant trophic group among nematodes^7^.

**Fig. 1:**
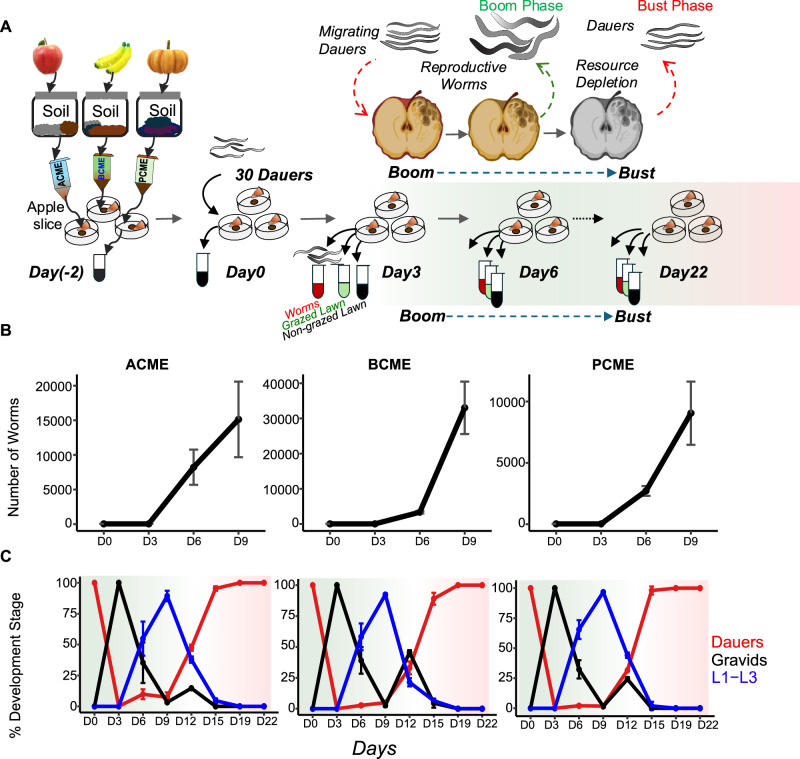
Population growth and changes in structure during the boom-to-bust cycle. **A** Schematic depiction of the *C. elegans* population boom-bust cycle and experimental design: preparation of compost microbial extracts (CMEs) and the sampling process. **B** Worm Population growth on different CMEs. Shown are averages ± SDs from three independent populations. Numbers were calculated from samples of harvested populations. **C** Percentage of worms of designated developmental stages in the population along the experiment progression. Shown are averages ± SDs for three replicates; *N* = 30–766 worms per time point, per graph (*N* was 30 worms at Day 0).

*C. elegans* is a popular model organism and the best-characterized nematode species, first introduced into molecular and developmental biology in 1974^8^, and later becoming the first multicellular organism to have its genome completely sequenced^9^. Like many other nematode species, the bacteriovore free-living *C. elegans* goes through population cycles of boom to bust. In nutrient-rich environments, such as on rotting fruit or other organic matter, worms reproduce through self-fertilization (primarily) and populations expand rapidly^10^. When nutrients are depleted, larvae arrest at the L1 stage or as dauer larvae. Dauers are stress-resistant, long-lived, and display behaviors such as nictation that increase the likelihood of being carried away on passing arthropods, dispersing to new environments and new sources of food, to resume development and reproduce (Fig. 1A)^11,12^. This dynamic allows *C. elegans* to persist in unpredictable and transient environments^13–15^.

*C. elegans* feeding behavior shows complex patterns of food preference associated with bacterial nutrient content and modulated by sensing of secreted molecules^16–19^. In addition, *C. elegans*, as other nematodes, harbors a characteristic and persistent gut microbiome, which contributes to its health and fitness^20–26^. Feeding and harboring bacteria may play important roles in shaping the worm’s bacterial environment, and although recent studies have begun to explore these roles^24,27^ still little is known. Furthermore, it is yet unclear to what extent worms carry bacteria with them from one environment to another^28,29^. Nevertheless, the presence of large populations of worms grazing on bacteria and exchanging bacteria with the environment may have a significant impact on the microbial environment. To explore this possibility, we introduced dauer larvae to three different bacterial environments emulating the ecological complexity of decomposing habitats, while enabling control and reproducibility, and monitored microbiome composition in worms and in their environment as the worm population expanded. Starting with “migrating” dauers, we show that over a period of 3–4 generations, worm growth and grazing altered the respective microbial environments. Furthermore, the presence of worms led to some convergence of the microbial composition across initially different microbiome community structures, supporting a role for bacterivorous nematodes in reshaping their local environment. These findings highlight the bidirectional interactions between the host and its microbial environment and offer new insights into the significance of nematodes in their respective ecological niches.

## Results

### The boom-to-bust cycle of *C. elegans* in a laboratory setting

Worms were raised through several generations in environments emulating their natural habitat, with apple slices laid on agar plates and seeded with three different compost-derived microbial communities sustained by the apple-derived nutrients. Following 2 days for acclimatization, 30 dauer larvae were added to each plate, with respective control plates containing no worms (Fig. 1A). Worms, the bacterial lawns on which they grew, and control ungrazed lawns were harvested every three days. By day three of the experiment, dauers reached adulthood, started reproducing, and the population began expanding rapidly (Fig. 1B, Supplementary Table 2). By day nine, worm corpses appeared, and the fraction of larvae in the population reached its peak (Fig. 1C, Supplementary Table 2). While a minor increase in the fraction of adult gravid worms appeared after this day, for the most part, worms had stopped maturing, and increasingly more larvae were arrested as SDS-resistant dauers. By day 15, all populations consisted mostly of dauers. It is estimated that worm populations underwent about four generations before arresting as dauers.

### *C. elegans* contributes to shaping its microbial environment

**Fig. 2:**
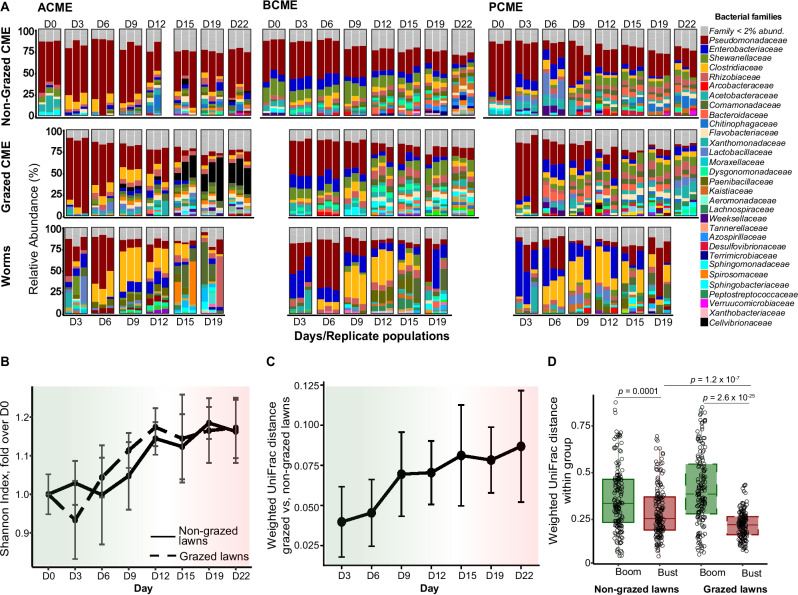
Changes in microbiome composition and diversity during the boom-to-bust cycle. **A** Microbiome composition in ungrazed and grazed lawns, and in worms raised on three different CMEs. Each stack plot represents microbiome composition in an independent plate/population (with 30 worms on day 3 and up to several thousands harvested at later time points), color-coded according to bacterial families. **B** Shannon diversity in lawn samples shown in (**A**). Shown are averages of fold values ± SDs, *N* = 9 per time point/curve. **C** Averages and SDs of all the pairwise distances between microbiomes in non-grazed and grazed lawns of the same CME and time point (*N* = 27 distances for each time point. **D** All pairwise distances among microbiomes of designated environments of boom (Days 3 and 6) and bust phase (Days 19 and Day 22), combining samples across all CME groups. *N* = 153 pairwise distances per group (18 samples). Boxplots show individual distances (their medians are represented by horizontal lines), 25th to 75th interquartile percentile range (boxes), and their maxima and minima (whiskers), Welch’s *t*-test.

To investigate the impact of *C. elegans* populations on their microbial environment, we performed next-generation bacterial 16S rRNA gene sequencing, yielding a detailed picture of microbiome composition in lawn environments prior to the addition of worms (Supplementary Fig. S2), and following worm addition - in lawns grazed by worms, in the gut of worms raised on these lawns, and in control non-grazed lawns (Fig. 2A). The microbial composition of the three compost-derived microbial communities started as very different (Supplementary Fig. S2): the apple-enriched CME (ACME) was dominated by members of the *Pseudomonadaceae, Aeromonadaceae,* and *Arcobacteriaceae* families; the banana-enriched CME (BCME) exhibited a microbiome primarily dominated by *Enterobacteriaceae* and *Arcobacteriaceae*; and the pumpkin-enriched CME (PCME) was dominated by *Moraxellaceae* and *Arcobacteriaceae*. Following 2 days on apple slices, compost-derived microbiomes maintained much of their differences, but were taken over by *Pseudomonadaceae* bacteria (Fig. 2A). This was the point when worms were added to some of the plates (day 0). Differences between CME communities without worms were maintained throughout the experiment (Supplementary Fig. S3).

Alpha diversity evaluated in lawn microbial environments from day 0 onward showed a similar increase with or without worms. This trend was similar to all diversity measures tested (Fig. 2B and Supplementary Fig. S4). However, beta diversity estimates showed that changes in microbiome composition in environments with or without worms followed different trajectories. The composition of worm-grazed lawns became increasingly dissimilar from that of non-grazed lawns over time, suggesting that the presence of worms had a significant effect on the microbial environment (Fig. 2C). In both control environments and in worm-grazed environments, distances between different CME microbiomes, which started as distinct, became smaller with time, suggesting convergence in community composition (Fig. 2D left). This might be due to the transition to a common substrate (apple) or due to ecological succession, in which strong competitors take over as nutrients are depleted. However, importantly, the presence of worms drove significantly more pronounced differences between early environmental microbiome and late ones, signifying worm-dependent convergence (Fig. 2D right and Supplementary Fig. S3).

**Fig. 3:**
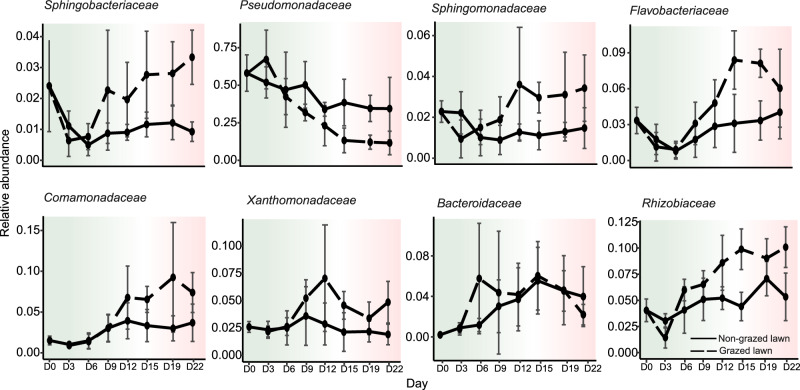
Bacterial families with differential abundances between grazed and non-grazed lawn environments. Shown are families with differential abundance adjusted for temporal effects. Lines represent mean relative abundance for each family within grazed (dashed lines) or non-grazed lawns (solid lines). Error bars extend one SD above and below each mean for that time point. Panels are sorted by Benjamini–Hochberg-adjusted *p*-value (FDR < 0.05) of the interaction effect, which reflects the degree to which grazing effects on that family vary over time, *N* = 9 samples per time point.

We next examined what may be the common denominator(s) making worm-grazed environments different from their non-grazed counterparts, and more similar to each other. We found that the most conspicuous of those was the depletion of *Pseudomonadaceae* bacteria (Figs. 2A, 3). On the other hand, *Comamonadaceae* family members became more abundant in worm-grazed environments, as well as several more minor members of the community—*Sphingobacteriaceae, Sphingomonadaceae, Flavobacteriaceae, Xanthomonadaceae,* and *Rhizobiaceae* (Fig. 3). Interestingly, these families, identified to be modulated by the presence of worms, were previously described as members of the *C. elegans* core gut microbiome^20,30^, suggesting that worm feeding on certain bacteria, and/or serving as a reservoir for bacterial proliferation and dissemination may affect bacterial environmental abundance.

### Worm gut microbiomes diverge by the end of the boom-to-bust cycle

**Fig. 4:**
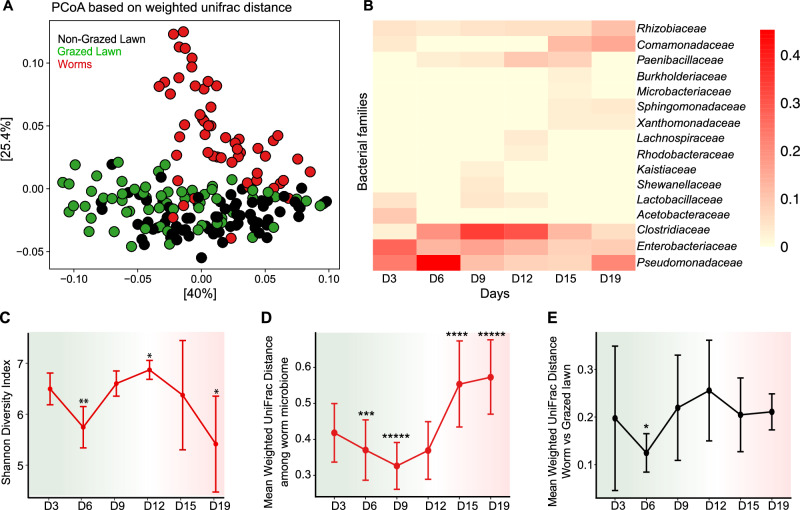
Worm gut microbiome composition diverges as populations approach the bust phase. **A** PCoA based on Weighted Unifrac distances, highlighting the differences between worm microbiomes and their microbial environments (*p* = 0.001, PERMANOVA). **B** Heatmap highlighting shared gut bacterial families along the course of the experiment. Families presented are those with a mean relative abundance ≥2% shared among all 9 communities (3 CMEs, 3 repeats each) for each time point. **C** Alpha diversity of worm gut microbiomes on different CMEs during the boom-to-bust progression. Shown are means ± SDs of nine independent populations per time point; statistical significance in diversity was assessed at each time point compared to day 3 using Welch’s *t*-test, **p < 0.001, *p < 0.01. **D** Weighted UniFrac distances among all worm gut microbiomes within the same time point. Data represent mean ± SD for *N* = 36 pairwise distances per time point. **E** Pairwise weighted UniFrac distances between worm microbiomes and grazed lawn environments within the same CME and time point. Data represent mean ± SD for *N* = 27 pairwise distances per time point, statistical significance was assessed at each time point compared to day 3 using Welch’s *t*-test, *p < 0.05.

To examine the relationship between environmental communities and the worm gut microbiome, we analyzed the sequencing data from worm samples. Previous work has shown that worms harbored a characteristic gut microbiome, distinct from their microbial environments but similar among worms raised in different environments^20,30^. This trend held true here as well (Fig. 4A).

Additional analysis identified the bacterial families that were present in worms in each of the time points, shared in all nine repeats across the different CMEs (Fig. 4B). Bacteria of most of these families were previously identified as core members of the *C. elegans* gut microbiome, with an addition of members of the *Clostridiaceae* family^20,30,31^. Bacterial families identified as part of the gut microbiome also seemed to be those affected in the environment by worm presence (Fig. 3), suggesting an association between worms feeding on, and harboring, certain bacteria, and the latter’s environmental abundance.

Additional examination of worm microbiomes identified three phases. The first was the transition from the pioneering generation to adults of the second generation (day 3 to day 6), which was accompanied by a significant decrease in gut microbial diversity (Fig. 4C, and Fig. 2A). Assuming that analyzed microbiomes in day 6 are dominated by the larger gut communities of second generation adults (while acknowledging some contribution from second generation larvae), the day 3 to day 6 shift may reflect a difference between gut microbiomes assembled in worms re-entering development after dauer arrest and those assembled in worms undergoing uninterrupted development. In the second phase, advancing into the boom phase (the first 12 days), microbial diversity in worms fluctuated (Fig. 4C), but gut microbiome composition across all CMEs was relatively similar (i.e., beta diversity was low, Fig. 4D), demonstrating a relatively stable composition. Subsequently, as dauers (which are largely devoid of bacteria^28^) became a majority, microbial diversity declined, and gut microbiome composition diverged (Fig. 4C, D). Notably, the differences in microbiome of worms remain distinct from their corresponding CMEs as time advances but did not show a clear pattern of development over time (Fig. 4E). Together, these results suggest that gut microbiomes during the boom phase (day 6 to day 12) are those that best represent the core gut microbiome, whereas the diverging worm communities observed during the bust phase may be affected by inter-population variation associated with food scarcity in the remaining larvae and adults^28^.

## Discussion

The experiment presented here examined the impact of nematode populations on their microbial environment in a setting that emulated the ecological complexity of decomposing habitats, yet enabled control, reproducibility, and quantitative monitoring. Our results demonstrate that *C. elegans* populations alter their microbial environment, and with it probably also the chemical constitution of this environment. Importantly, changing the environment appeared to be deterministic to some degree, as worm presence altered environmental communities that began as distinct to become more similar to each other, with certain bacterial families reproducibly depleted and others reproducibly enriched. While it is tempting to consider this process as niche engineering, the significance of the apparent microbiome convergence for worm fitness is yet unknown. Nevertheless, there appear to be some clear winners, which are the bacteria that become more enriched thanks to the worm presence.

Among the most conspicuous worm-associated changes in the environment, we observed the depletion of *Pseudomonadaceae* family members. While bacteria of this family are dominant members of the worm gut microbiome, their depletion in the environment went hand in hand with lower abundance in the worm gut. Previous work suggested that worms (early gravid adults) were able to enrich for these bacteria in their gut even if their environmental abundance was low^20^. The current results suggest that as worms spend more time in environments and as their age structure changes, this relationship may not hold, leading to gut abundance becoming more correlated with environmental availability. On the other hand, *Comamonadaceae, Sphingobacteriaceae, Sphingomonadaceae, Flavobacteriaceae, Xanthomonadaceae,* and *Rhizobiaceae* family members became more enriched in worm-grazed environments. *Flavobacteriaceae* and *Sphingobacteriaceae* bacteria were not found to be a significant part of the worm gut microbiome in the current study due to relatively low gut abundance, but were described elsewhere to be part of the worm gut microbiome^20,30,32^. Enrichment of worm gut microbiome members in worm-grazed environments suggests that worms may serve as a reservoir for these bacteria, seeding the environment.

Most animals harbor microbial communities that contribute to vital functions such as digestion, immunity, and development. The composition and assembly of these microbiomes are often influenced by the surrounding environment. This has been shown in insects^33^, saltwater and freshwater fish^34^, and birds^35^. Our findings highlight a more reciprocal relationship in which the host also affects the composition of the environmental microbial pool. Our results provide evidence that nematodes such as *C. elegans* play an important role in modifying microbial ecosystems as part of their population growth (and collapse).

In previous work, we showed that dauer larvae, which migrate and colonize new niches, are largely devoid of gut bacteria and cannot disseminate microbes across environments^28^. More recent results suggest that specific gut bacteria, of the *Lelliotia* genus, may persist in dauers and travel with them to new niches^29^. The results presented here show that whether carrying bacteria or not, colonizing dauers can re-establish the worm gut microbiome, and along the way change their environment.

While nematodes are generally considered important for nutrient recycling, relatively little is known about how exactly they do that. Our findings with the bacterivore *C. elegans* demonstrate that one such mechanism could be through their effects on environmental bacterial abundance, which in turn will affect nutrient availability. Altogether, our findings present *C. elegans* and its microbiome both as affected by the microbial environment and as a modulator of the microbial environment and the ecosystem as a whole.

## Methods

### Worm growth

Wildtype N2 worms, originally obtained from the *Caenorhabditis* Genome Center, were used in all experiments and raised at 20 °C. Dauer used to initiate experiments were obtained from worms raised on nematode growth medium (NGM) plates seeded with *E. coli* OP50 bacteria for several generations. To eliminate non-dauers, harvested worms were incubated with 1% sodium dodecyl sulfate (SDS), gently rotating on an orbital shaker for 25 min^14^. SDS-resistant dauers were washed several times in M9 salt solution to remove SDS and, along with debris from lysed worms, pipetted onto the center of a sterile 2% agar plate. Following dauer dispersal throughout the plate, the center part of the agar, with the remaining debris (but without worms), was cut and removed, and the dispersed dauers were washed off and resuspended in M9.

### Compost microbial extracts (CMEs)

CMEs offering natural-like microbial diversity were prepared as previously described^28,36^. Briefly, compost was prepared from soils collected from 3 different locations around campus, enriched by supplementing with apple slices (ACME), banana peels (BCME), or pumpkin slices (PCME), and incubated at room temperature for 2–3 weeks to allow development of distinct microbial communities. CMEs were prepared from compost batches by resuspension in M9, centrifugation in a clinical centrifuge at 1800 rpm to pellet particulate matter (and if existing, native soil worms), and filtering supernatants through two layers of tissue paper to remove smaller particles. Filtrates were collected in aliquots in Eppendorf tubes, concentrated by pelleting on a microcentrifuge at maximum speed for 2 min, resuspended in 200 µL M9, and kept in the fridge for no more than a week. Prior to experiments, the efficacy of the CMEs in supporting *C. elegans* development to adulthood was tested.

### Design of boom-to-bust experiments

Thirty dauer larvae were added to each of 60-mm diameter plates on which a 3-mm thick apple slice was placed (surface sterilized with 70% ethanol) and seeded 2 days earlier with 200 µL CME (Fig. 1A). Apples were used as a source of nutrients to support microbial communities over the course of the experiment, while maintaining structural integrity. Twenty-four replicate plates in total were set for each of the three different CMEs so that three replicate populations could be harvested for each time point. Worm populations were allowed to grow continuously without bacterial supplementation up to 22 days (~4 generations), and the entire microbial community and worm population were harvested from each plate at eight time points, as designated.

Control plate sets were prepared with the same CMEs to which no worms were added, used to harvest plate microbial communities at the same time points as those of the experimental plates, and representing non-grazed environments.

### Sampling environmental microbial communities and worm populations

From each sampled plate, the microbial lawn with the apple slice and the entire worm population were suspended in M9, scraped, and washed off into a 15 ml conical tube. Apple slices were removed, worms were spun down by light centrifugation on a clinical centrifuge at 1800 rpm, and the supernatant with bacteria of the worm-grazed lawn was removed to a separate tube. The pellet, containing both worms and bacteria, was pipetted onto the center of a sterile 100 mm 2% agar plate, allowing worms to disperse. Dead worms were manually removed from the center, and an agar slab with the remaining bacteria was cut and washed into the tube containing the rest of the bacterial lawn. This tube was centrifuged at maximal speed for 10 min to pellet bacteria, and the pellet was stored at −20 °C awaiting DNA extraction. Worms were washed off the agar plate and collected for counting and for DNA extraction from worms and their gut bacteria. A 100 μL sample was taken for counting worms, estimating population size, and distribution between developmental stages. Another 100 μL sample was treated with 1% SDS treatment, to estimate the proportion of SDS-resistant dauers^5,28^. Worms remaining in the main batch were washed and surface sterilized for DNA extraction. Briefly, worms were washed three times with M9 + Triton, paralyzed with 10 mM levamisole to prevent pharyngeal pumping, and surface-sterilized with 2% bleach in M9^31^. Worms were washed twice with sterile M9 to remove bleach and stored at −20 °C until DNA extraction.

Ungrazed lawns were harvested using M9 buffer, centrifuged at high speed for 10 min, and the resulting bacterial pellet was stored at −20 °C until DNA extraction.

### DNA extraction for 16S rRNA gene sequencing

DNA was extracted from worm and lawn samples as previously described, using the DNeasy PowerSoil Pro Kit (Qiagen Cat. #47016) and eluted in 60 μl elution buffer^37^. DNA extracted from worm and lawn samples served as template in PCR reactions with primers 515 F and 806 R and with Illumina Nextera XT DNA Library Preparation Kit (Cat. #FC-131-1096) to generate sequencing amplicon libraries of the bacterial 16S V4 region, as previously described^28^. Paired-end sequencing was performed on an in-house Illumina Miniseq platform using the High-Output (300 cycles) Illumina kit (Cat. #FC-420-1003). Control libraries generated from reagents used for DNA extraction and library preparation (instead of the DNA template) were also prepared to assess the contribution of bacterial DNA contamination to the results.

### Microbiome data processing and exploratory analysis

Sequencing yielded high-quality data (Q30) with an average of 183607.2 ± 161662.4 reads per sample (Supplementary Table 1). FASTQ files were processed using DADA2 v1.32^38^, including estimation of error models (independently for forward and reverse reads), denoising, merging of paired-end reads, and removal of chimeras (using the Consensus method) to generate a table of amplicon sequence variants (ASVs) containing an average of 105,998.2 ± 103,092.1 reads per sample. Subsequently, ASVs with fewer than 200 total reads across analyzed samples were excluded. Taxonomic assignments were carried out using the SILVA database^39^ (v138.1). The final ASV table, merged with the corresponding metadata and taxonomy files were imported into R using phyloseq v1.50.0^40^ for downstream analyses. Raw data is available in the NCBI SRA database with accession number PRJNA1116742, and the Phyloseq object is provided as Supplementary File 1.

Alpha Diversity was calculated using the *plot_richness()* function in phyloseq. To normalize diversity across the different CMEs, indices for each replicate at each time point within a given CME were divided by the average diversity index for the respective CME at day 0.

Beta diversity estimates used Weighted UniFrac distances, computed based on ASV-level relative abundance and phylogenetic tree data using the distance() function in phyloseq. The phylogenetic tree was constructed by aligning sequences with DECIPHER^41^ and building a neighbor-joining tree using phangorn^42^.

Stacked bar plots were generated using relative abundance data aggregated at the bacterial family level to visualize community composition across samples.

SourceTracker analysis was employed to assess the contribution of microbial contaminants from reagents in the results obtained from experimental samples^43^. Sequencing of control samples containing reagents demonstrated no contribution to the analyzed data (Supplementary Fig. S1).

Graphs were drawn using the ggplot2 R package^44^.

### Statistical analyses

Differential abundance analysis was performed using bacterial family-level counts modeled with a beta-binomial distribution^45^, as implemented in the corncob R package (v0.4.2)^46^. Included in the analysis were only families with overall relative abundance above 1% in more than 75% of samples. Covariates considered in calculating significance were time and worm presence, and the interaction between the two. *P*-values were obtained for each covariate using the Wald test and merged using the Cauchy Combination rule^47^, and corrected for multiple testing with the Benjamini-Hochberg procedure^48^ (FDR < 0.05). Detailed description of the analysis can be found in Supplementary File 2.

Statistical differences in weighted UniFrac distances between grazed and non-grazed lawns were tested using Welch’s *t*-test. R-scripts used to generate figures are available at https://rpubs.com/MicroRB/Boom_Bust.

## Supplementary information

41522_2026_975_MOESM1_ESM

41522_2026_975_MOESM2_ESM

41522_2026_975_MOESM3_ESM

## Data Availability

Raw data is available in the NCBI SRA database with accession number PRJNA1116742.

## Code availability

R-scripts used to generate figures are available at https://rpubs.com/MicroRB/Boom_Bust.

## References

[1] Steel, H. et al. Nematode succession during composting and the potential of the nematode community as an indicator of compost maturity. *Pedobiologia (Jena).* **53**, 181–190 (2010).

[2] Hodda, M. Phylum Nematoda: a classification, catalogue and index of valid genera, with a census of valid species. *Zootaxa.* **5114**, 1–289 (2022). 35391386 10.11646/zootaxa.5114.1.1

[3] Zhang, C., Wright, I. J., Nielsen, U. N., Geisen, S. & Liu, M. Linking nematodes and ecosystem function: a trait-based framework. *Trends Ecol. Evol.* **39**, 644–653 (2024). 38423842 10.1016/j.tree.2024.02.002

[4] Vlaar, L. E. et al. On the role of dauer in the adaptation of nematodes to a parasitic lifestyle. *Parasit. Vectors* **14**, 10.1186/S13071-021-04953-6 (2021). 10.1186/s13071-021-04953-6 PMC8555053 34706780

[5] Hu, P. J. Dauer. in *WormBook**: The Online Review of C. elegans Biology.*(WormBook, 2007) 1–19. 10.1895/wormbook.1.144.1. 10.1895/wormbook.1.144.1 PMC2890228 17988074

[6] Hand, S. C., Denlinger, D. L., Podrabsky, J. E. & Roy, R. Mechanisms of animal diapause: recent developments from nematodes, crustaceans, insects, and fish. *Am. J. Physiol. Regul. Integr. Comp. Physiol.* **310**, R1193–R1211 (2016). 27053646 10.1152/ajpregu.00250.2015 PMC4935499

[7] van den Hoogen, J. et al. Soil nematode abundance and functional group composition at a global scale. *Nature* **572**, 194–198 (2019). 31341281 10.1038/s41586-019-1418-6

[8] Brenner, S. The genetics of *Caenorhabditis elegans*. *Genetics* **77**, 71–94 (1974). 4366476 10.1093/genetics/77.1.71 PMC1213120

[9] Genome sequence of the nematode *C. elegans*: a platform for investigating biology. *Science* **282**, 2012–2018 (1998). 10.1126/science.282.5396.2012 9851916

[10] Kiontke, K. & Sudhaus, W. Ecology of Caenorhabditis species. *WormBook*10.1895/wormbook.1.37.1 (2006). 10.1895/wormbook.1.37.1 PMC4780885 18050464

[11] Petersen, C. et al. Travelling at a slug’s pace: possible invertebrate vectors of *Caenorhabditis nematodes*. *BMC Ecol.* **15**, 19 (2015). 26170141 10.1186/s12898-015-0050-z PMC4501285

[12] Reed, E. M. & Wallace, H. R. Leaping locomotion by an Insect-parasitic Nematode. *Nature.* **206**, 210–211 (1965).

[13] Cassada, R. C. & Russell, R. L. The dauerlarva, a post-embryonic developmental variant of the nematode *Caenorhabditis elegans*. *Dev. Biol.* **46**, 326–342 (1975). 1183723 10.1016/0012-1606(75)90109-8

[14] Androwski, R. J., Flatt, K. M. & Schroeder, N. E. Phenotypic plasticity and remodeling in the stress-induced Caenorhabditis elegans dauer. Wiley Interdiscip. *Rev Dev. Biol.* **6**, e278 (2017). 10.1002/wdev.278 PMC5626018 28544390

[15] Félix, M. A. & Duveau, F. Population dynamics and habitat sharing of natural populations of Caenorhabditis elegans and C. briggsae. *BMC Biol.* **10**, 59 (2012). 22731941 10.1186/1741-7007-10-59 PMC3414772

[16] Dallière, N., Holden-Dye, L., Dillon, J., O’Connor, V. & Walker, R. J. *Caenorhabditis elegans* feeding behaviors. *Oxf. Res. Encycl. Neurosci.*10.1093/acrefore/9780190264086.013.190 (2017).

[17] Pérez-Carrascal, O. M. et al. Host preference of beneficial commensals in a microbially-diverse environment. *Front. Cell. Infect. Microbiol.* **12**, 795343 (2022). 35782135 10.3389/fcimb.2022.795343 PMC9240469

[18] Siddiqui, R. et al. Olfactory basis for essential amino acid perception during foraging in *Caenorhabditis elegans*. *Elife* **13**, RP101936 (2024).

[19] García-González, A. P. et al. Bacterial metabolism affects the *C. elegans* response to cancer chemotherapeutics. *Cell* **169**, 431–441.e8 (2017). 28431244 10.1016/j.cell.2017.03.046 PMC5484065

[20] Berg, M. et al. Assembly of the *Caenorhabditis elegans* gut microbiota from diverse soil microbial environments. *ISME J.* **10**, 1998–2009 (2016). 26800234 10.1038/ismej.2015.253 PMC5029150

[21] Meyer, J. M. et al. Succession and dynamics of *Pristionchus nematodes* and their microbiome during decomposition of *Oryctes borbonicus* on La Réunion Island. *Environ. Microbiol.* **19**, 1476–1489 (2017). 28198090 10.1111/1462-2920.13697

[22] Zhang, F. et al. Natural genetic variation drives microbiome selection in the Caenorhabditis elegans gut. *Curr. Biol.* **31**, 2603–2618.e9 (2021). 34048707 10.1016/j.cub.2021.04.046 PMC8222194

[23] Griem-Krey, H., Petersen, C., Hamerich, I. K. & Schulenburg, H. The intricate triangular interaction between protective microbe, pathogen and host determines fitness of the metaorganism. *Proc. Biol. Sci.* **290**, 20232193 (2023). 38052248 10.1098/rspb.2023.2193 PMC10697802

[24] Lo, W. S., Sommer, R. J. & Han, Z. Microbiota succession influences nematode physiology in a beetle microcosm ecosystem. *Nat. Commun.* **15**, 5137 (2024). 38879542 10.1038/s41467-024-49513-5 PMC11180206

[25] Peters, L. et al. Polyketide synthase-derived sphingolipids mediate microbiota protection against a bacterial pathogen in C. elegans. *Nat. Commun.* **16**, 5151 (2025). 40461452 10.1038/s41467-025-60234-1 PMC12134224

[26] Berg, M., Zhou, X. Y. & Shapira, M. Host-specific functional significance of Caenorhabditis gut commensals. *Front. Microbiol.* **7**, 221536 (2016). 10.3389/fmicb.2016.01622 PMC5066524 27799924

[27] Johnke, J. et al. Caenorhabditis nematodes influence microbiome and metabolome characteristics of their natural apple substrates over time. *mSystems* **10**, e0153324 (2025). 39791908 10.1128/msystems.01533-24 PMC11834410

[28] Bodkhe, R., Trang, K., Hammond, S., Jung, D. K. & Shapira, M. Emergence of dauer larvae in *Caenorhabditis elegansdisrupts* continuity of host-microbiome interactions. *FEMS Microbiol. Ecol.* **100**, fiae149 (2024). 39516048 10.1093/femsec/fiae149 PMC11590253

[29] Rivera, D. E. et al. Dynamics of gut colonization by commensal and pathogenic bacteria that attach to the intestinal epithelium. *NPJ Biofilms Microbiomes* **11**, 70 (2025). 40319018 10.1038/s41522-025-00696-9 PMC12049552

[30] Zhang, F. et al. *Caenorhabditis elegans* as a model for microbiome research. *Front. Microbiol.* **8**, 241616 (2017). 10.3389/fmicb.2017.00485 PMC5362939 28386252

[31] Dirksen, P. et al. The native microbiome of the nematode Caenorhabditis elegans: gateway to a new host-microbiome model. *BMC Biol.* **14**, 38 (2016). 27160191 10.1186/s12915-016-0258-1 PMC4860760

[32] Dirksen, P. et al. CeMbio—the *Caenorhabditis elegans* microbiome resource. *G3 GenesGenomesGenetics* **10**, 3025 (2020). 10.1534/g3.120.401309 PMC7466993 32669368

[33] Yun, J. H. et al. Insect gut bacterial diversity determined by environmental habitat, diet, developmental stage, and phylogeny of host. *Appl. Environ. Microbiol.* **80**, 5254 (2014). 24928884 10.1128/AEM.01226-14 PMC4136111

[34] Sullam, K. E. et al. Environmental and ecological factors that shape the gut bacterial communities of fish: a meta-analysis. *Mol. Ecol*. 10.1111/j.1365-294X.2012.05552.x (2012). 10.1111/j.1365-294X.2012.05552.x PMC3882143 22486918

[35] Liukkonen, M. et al. Seasonal and environmental factors contribute to the variation in the gut microbiome: a large-scale study of a small bird. *J. Anim. Ecol.* **93**, 1475–1492 (2024). 39041321 10.1111/1365-2656.14153

[36] Trang, K., Bodkhe, R. & Shapira, M. Compost microcosms as microbially diverse, natural-like environments for microbiome research in *Caenorhabditis elegans*. *J. Vis. Exp* **187**, 64393 (2022). 10.3791/64393 PMC9576343 36190292

[37] Choi, R. et al. An enterobacteriaceae bloom in aging animals is restrained by the gut microbiome. *Aging Biol.* **1**, 20240024 (2024). 10.59368/agingbio.20240024 PMC11085993 38736850

[38] Callahan, B. J. et al. DADA2: high-resolution sample inference from Illumina amplicon data. *Nat. Methods* **13**, 581–583 (2016). 27214047 10.1038/nmeth.3869 PMC4927377

[39] Quast, C. et al. The SILVA ribosomal RNA gene database project: improved data processing and web-based tools. *Nucleic Acids Res.* **41**, D590–D596 (2013). 23193283 10.1093/nar/gks1219 PMC3531112

[40] McMurdie, P. J. & Holmes, S. *phyloseq*: an R package for reproducible interactive analysis and graphics of microbiome census data. *PLoS ONE* **8**, e61217 (2013). 23630581 10.1371/journal.pone.0061217 PMC3632530

[41] Wright, E. S. Fast and flexible search for homologous biological sequences with DECIPHER v3. *R. J.* **16**, 191–200 (2024).

[42] Schliep, K. P. phangorn: phylogenetic analysis in R. *Bioinformatics* **27**, 592 (2010). 21169378 10.1093/bioinformatics/btq706 PMC3035803

[43] Knights, D. et al. Bayesian community-wide culture-independent microbial source tracking. *Nat. Methods* **8**, 761–763 (2011). 21765408 10.1038/nmeth.1650 PMC3791591

[44] Wickham, H. *ggplot2*. 10.1007/978-3-319-24277-4 (2016).

[45] Martin, B. D., Witten, D. & Willis, A. D. Modeling microbial abundances and dysbiosis with beta-binomial regression. *Ann. Appl. Stat.* **14**, 94–115 (2019). 10.1214/19-aoas1283 PMC7514055 32983313

[46] Martin, B. D., Witten, D. & Willis, A. D. corncob: Count Regression for Correlated Observations with the Beta-Binomial. *CRAN: Contributed Packages*10.32614/CRAN.package.concorb (2021).

[47] Liu, Y. & Xie, J. Cauchy combination test: a powerful test with analytic p-value calculation under arbitrary dependency structures. *J. Am. Stat. Assoc.* **115**, 393 (2019). 33012899 10.1080/01621459.2018.1554485 PMC7531765

[48] Benjamini, Y. & Hochberg, Y. Controlling the false discovery rate: a practical and powerful approach to multiple testing. *J. R. Stat. Soc.* **57**, 289–300 (1995).

