# Supplementary material for: Caenorhabditis elegans populations shape their microbial environment
Source: NPJ Biofilms Microbiomes. 2026 Apr 4;12:106. doi: 10.1038/s41522-026-00975-z (PMC13230874; doi:10.1038/s41522-026-00975-z)
Supplement: Supplementary file 1 — 41522_2026_975_MOESM1_ESM [file 41522_2026_975_MOESM1_ESM.pdf]

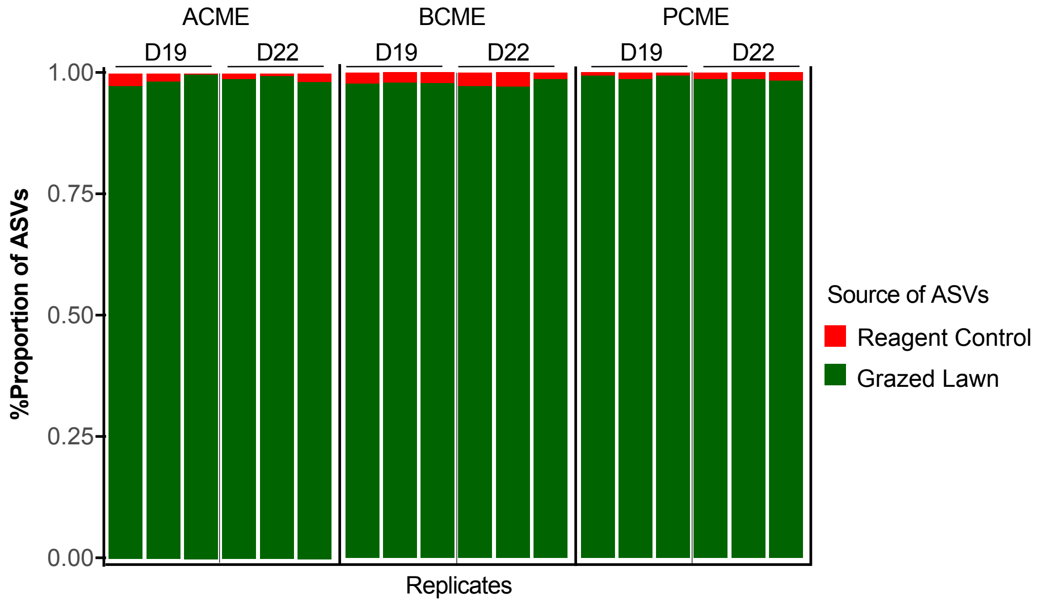


**Supplementary Figure S1. No contribution of reagent-derived contaminants confirms the biological origin of microbial DNA in grazed lawn samples.** Bar plots show SourceTracker-estimated proportions of sequences in each sample attributed to control sources (DNA extraction and library preparation reagents alone; "Control") versus grazed lawn sources.

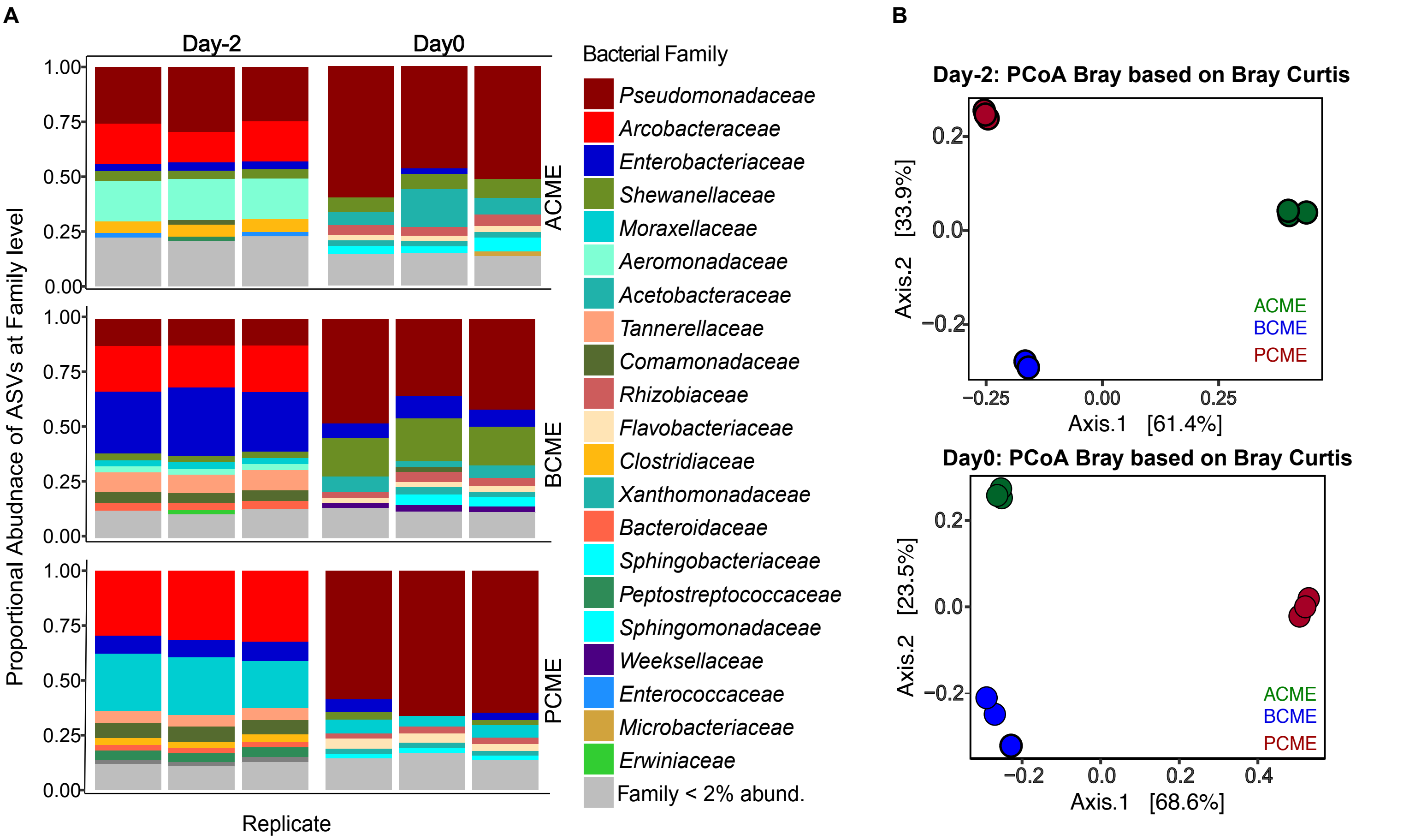


**Supplementary Figure S2. Combining different soils and produce results in distinct microbial communities that dauers were exposed to. A.** Stacked bar plots showing the relative abundance of ASVs at the family level. **B.** PCoA based on Bray-Curtis distance illustrating microbiome composition in different CMEs before (Day -2) and after (Day 0) the addition of produce. While Pseudomonadaceae abundance increased across all CMEs following the introduction of an apple slice, overall microbiome composition differed significantly (Day -2, p = 0.004; Day 0, p = 0.004; PERMANOVA).

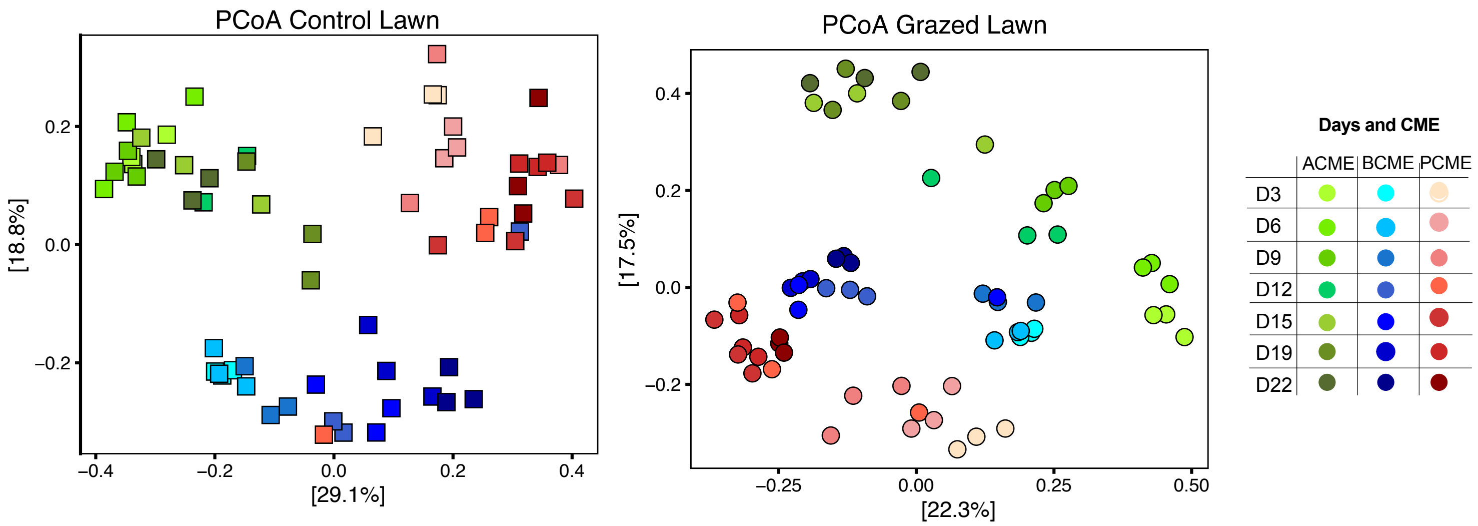


**Supplementary Figure S3. Non-grazed lawn microbiomes remain distinct over the course of the experiment while worm-grazed microbiomes converge. A.** PCoA for microbiomes ofnon-grazed lawn environments or of **B.** worm-grazed lawn environments **.** Note the distinct clusters of CME microbiomes marked with similar colors (red, blue or green) for non-grazed communities, in contrast to a shift to the left for darker shades among worm-grazed communities.

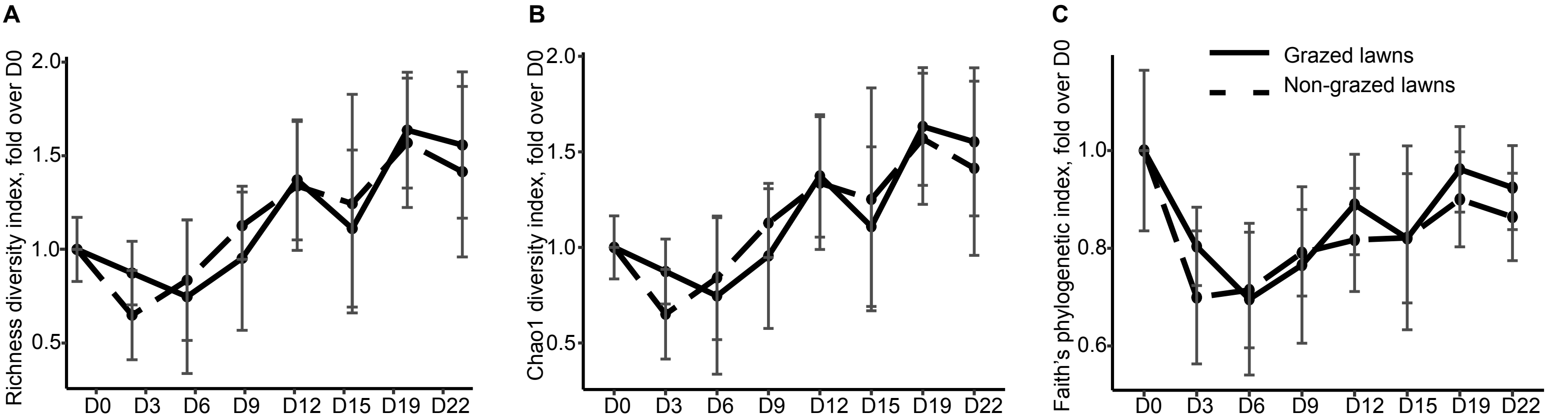


**Supplementary Figure S4. Alpha diversity in grazed and non-grazed lawns. A**. Observed richness, **B**. Chao1 index, **C**. Faith's Phylogenetic Diversity index. Data represent mean ± standard deviation (SD); N = 9 per group per time point.

**Supplementary file 2**
**Differential Abundance and Variability Analysis**

We applied a beta-binomial regression model[1] as implemented in corncob[2] v0.4.2 to test differential abundance and variability of bacterial families in response to *C. elegans* grazing. This model assumes that the abundance $y_{ij}$ of family *j* in sample *i* follows a Binomial distribution with Beta-distributed probability:

$$y_{ij} | p_{ij} \sim \text{Bin}\left(N_i, p_{ij}\right) \quad (1)$$

$$p_{ij} \sim \text{Beta}\left(\mu_{ij}, \varphi_{ij}\right), \quad (2)$$

where $N_i$ is the read depth of sample *i*. The parameters $\mu_{ij}$ and $\varphi_{ij}$ represent the mean and dispersion for family *j* in sample *i*, using the mean-dispersion parameterization of the Beta distribution. Whenever $\varphi_{ij} > 0$, this approach supports overdispersion, the phenomenon where the variance of $y_{ij}$ exceeds what would be expected in a Binomial distribution. This allows for testing of both differential abundance (changes in mean) and variability (change in dispersion) as a function of experimental factors.

To relate experimental factors with model parameters, we define a covariate vector that reflects worm presence, temporal effects, and their interaction:

$$x_i^\top = \begin{bmatrix} 1 \\ \mathbb{I}\left(i \in \text{Worms}\right) \\ \mathbb{I}\left(i \in (\text{Day } 7, \text{Day } 14]\right) \\ \mathbb{I}\left(i \in (\text{Day } 14, \text{Day } 22]\right) \\ \mathbb{I}\left(i \in (\text{Day } 7, \text{Day } 14]\right)\mathbb{I}\left(i \in \text{Worms}\right) \\ \mathbb{I}\left(i \in (\text{Day } 14, \text{Day } 22]\right)\mathbb{I}\left(i \in \text{Worms}\right) \end{bmatrix}. \quad (3)$$

where $\mathbb{I}()$ denotes the indicator function. These are linked to the mean and dispersion parameter through logistic transformations:

$$\text{logit}\left(\mu_{ij}\right) = x_i^\top \beta_j \quad (4)$$

$$\text{logit}\left(\varphi_{ij}\right) = x_i^\top \gamma_j. \quad (5)$$

The $\beta_j$ coefficients capture family *j*'s differential abundance effects associated with each input experimental factor. The $\gamma_j$ capture the analogous differential variability effects. The *C. elegans* * Time interaction terms (coefficients 5 and 6) identify temporal effects specific to the grazing condition.
For each family, the model above provides *p*-values for individual coefficients using a Wald test. To aggregate evidence across time windows, we apply the Cauchy combination rule[3]. For differential abundance, we merged *p*-values across $\beta_2$, $\beta_5$, and $\beta_6$. For differential variability, we merged *p*-values across $\gamma_2$, $\gamma_5$, and $\gamma_6$. Including only these terms identifies grazing-related shifts while adjusting for temporal effects in control samples. The resulting Cauchy-combined *p*-values were adjusted for multiple testing using the Benjamini-Hochberg (BH) false discovery rate procedure at a significance threshold of FDR < 0.05.

1. Martin BD, Witten D, Willis AD. MODELING MICROBIAL ABUNDANCES AND DYSBIOSIS WITH BETA-BINOMIAL REGRESSION. *Ann Appl Stat* 2020;**14**:94.
2. Martin BD, Witten D, Willis AD. corncob: Count Regression for Correlated Observations with the Beta-Binomial. *CRAN: Contributed Packages* 2021, DOI: 10.32614/CRAN.PACKAGE.CORNCOB.
3. Liu Y, Xie J. Cauchy combination test: a powerful test with analytic p-value calculation under arbitrary dependency structures. *J Am Stat Assoc* 2019;**115**:393.

---

**Supplementary Table 1.** Sequencing read retention across sequential microbiome processing steps.

**Supplementary Table 2.** Quantitative worm dynamics during the boom-to-bust transition.
